# Management of idiopathic granulomatous mastitis in lactation: case report and review of the literature

**DOI:** 10.1186/s13006-021-00370-8

**Published:** 2021-03-04

**Authors:** Hannah W. Kornfeld, Katrina B. Mitchell

**Affiliations:** 1 Santa Barbara Cottage Hospital, 400 W. Pueblo Street, Santa Barbara, CA 93105 USA; 2 Ridley Tree Cancer Center at Sansum Clinic, 540 West Pueblo Street, Santa Barbara, CA 93105 USA

**Keywords:** Breastfeeding, Idiopathic granulomatous mastitis, Triamcinolone, Lactation, Single breast lactation, Steroid injections, Lactation problems, Imaging, Postpartum, Infection, Inflammation

## Abstract

**Background:**

Idiopathic Granulomatous Mastitis (IGM) is a benign chronic inflammatory breast condition that mimics two common breast disorders: breast carcinoma and breast abscess. It can form breast masses, fistulae, and fluid collections, resulting in breast disfigurement with retraction and nipple areolar complex (NAC) inversion. IGM most often presents in women of childbearing age within a few years of pregnancy, and can significantly impact lactation. Despite the prevalence of this disease, no current literature describes an approach to managing IGM during breastfeeding.

**Case presentation:**

A 28-year-old G3P2 patient of Native American origin presented to her obstetrician at 7 months pregnant with worsening left breast swelling and redness. She underwent a mammogram, ultrasound and core needle biopsy that confirmed the diagnosis of Idiopathic Granulomatous Mastitis. During the postpartum period, she underwent intralesional triamcinolone injections of her left breast. Due to the contraindication of breastfeeding after local steroid injection, the patient stopped breastfeeding from the affected breast and continued breastfeeding unilaterally.

**Conclusions:**

Idiopathic Granulomatous Mastitis is a challenging chronic inflammatory breast disease that affects women primarily in the reproductive years, with a higher incidence in patients of Hispanic, Native American, Middle Eastern, and African descent. Treatment of IGM during pregnancy and lactation has thus far not been addressed. We review the literature on the treatment of IGM in the non-lactating population, and propose considerations for treating breastfeeding women affected by this disease. Traditional treatment has included systemic immunosuppression and surgery, but newer literature demonstrates that intralesional injection of steroid can provide significant symptomatic relief to patients. A diagnosis of IGM does not preclude breastfeeding, though patients may experience challenges with milk production and latch on the affected breast. Individualized care should be provided, with considerations given to the following: side effects of systemic steroids, the need to wean a breast being treated with intralesional steroids, and augmentation of milk production on the unaffected breast to promote continued breastfeeding.

## Background

Idiopathic Granulomatous Mastitis (IGM) is a benign chronic inflammatory breast condition, first described in 1972 by Kessler and Wooloch, that mimics two common breast disorders: breast carcinoma and breast abscess [1]. It can form breast masses, fistulae, and fluid collections, resulting in breast disfigurement with retraction and nipple areolar complex (NAC) and inversion. IGM most often presents in women of childbearing age within a few years of pregnancy, and is most common in women of Hispanic, Asian, Middle Eastern or African origin [2, 3]. Evaluation involves breast imaging with ultrasound and mammogram, and core needle biopsy to establish a diagnosis [4].

In the past, treatment of IGM involved incision and drainage of fluid collections, extensive debridement, mastectomy, as well as oral steroids, methotrexate and other anti-inflammatories including colchicine [5–7]. More recent management has shifted to minimally invasive approaches, including aspiration of fluid and intralesional injection of steroids [8, 9]. Additionally, methotrexate and azathioprine can be used as steroid-sparing agents and help promote long-term remission [10].

There is no consensus in the field regarding optimal management, even in non-lactating patients. Some of the uncertainty in treatment might come from uncertainty in causation. IGM has been thought of as infectious, inflammatory and autoimmune [11]. Recently, a few studies using low dose prednisone, 25 mg/day and 0.8 mg/kg/day have been shown effective in treatment of disease, supporting a more inflammatory or autoimmune etiology [12–14]. There is some suggestion that *Corynebacterium kroppenstedtii* is associated with IGM; however, there is no evidence that targeting these bacteria alters the disease course and may reflect normal breast microbiome [15, 16].

Treatment during breastfeeding poses a particular challenge in terms of managing symptoms and protecting lactation, though no current literature describes the approach to this clinical scenario. We describe the experience of a patient diagnosed during pregnancy with IGM, and her management during lactation. We review the literature on the treatment of IGM in the non-lactating population, and propose guidelines for treating breastfeeding women affected by this disease.

## Case presentation

A 28-year-old G3P2 patient of Native American origin presented to her obstetrician at 7 months pregnant with several months of left breast swelling and redness. *At the recommendation of her obstetrician,* she underwent diagnostic mammogram and ultrasound showing an ill-defined hypoechoic mass-like area in the left breast 10:00 position, 4 cm from the nipple. Core needle biopsy demonstrated granulomatous mastitis. She also had developed erythema nodosum on her bilateral lower extremities during pregnancy. Tuberculosis was ruled out. Evaluation for autoimmune disease, including serology, was negative. Her obstetrician started a steroid taper at 30 mg per day for a 6 day course, decreasing by 5 mg per day. The patient experienced improvement in pain and swelling during this time. This taper concluded 3 weeks prior to delivery; no further steroids were prescribed in order prevent hematologic or infectious complications at birth.

The patient began breastfeeding after delivery and continued to breastfeed from both breasts. She was not able to utilize an electric pump to express milk from her left breast due to discomfort and minimal milk extraction. She did report that the baby latched comfortably with audible swallows from the left breast, but she felt there was less milk production than from the right breast. The patient also felt she produced less milk in comparison to previously breastfeeding her other two children for 1 year without requiring infant formula. Beginning at 2 weeks postpartum, at the recommendation of the pediatrician, the patient initiated infant formula to maintain appropriate weight gain in the current infant. The infant had regained birthweight at two and a half weeks post- partum. Due to her left breast pain and swelling, the patient also restarted prednisone at 20 mg by mouth daily at 2 weeks postpartum.

She presented at 4 weeks postpartum to the breast surgeon for evaluation of worsening redness and pain in the left breast despite being compliant with prednisone. She noticed increased difficulty with her infant’s latch on the affected breast due to pain, as well as decreased swallows that she felt corresponded to decreased breastmilk volume. On exam, a large mass-like area with erythema and induration in the left breast was appreciated. This extended from the 9:00 to 4:00 position in the periareolar region, measuring approximately 8.0 × 8.0 × 9.0 cm total size. There was no fistula present.

The patient elected to undergo tapered discontinuation of her oral steroid over the course of 1 week and injection of 40 mg/1 mL triamcinolone mixed with 3 mL 2% lidocaine into the affected areas of her left breast. Due to long-acting depot mechanism and high transfer of triamcinolone into breastmilk, the breast surgeon advised her to discontinue breastfeeding from the affected breast; triamcinolone was expected to remain in the breast for 3 weeks until the time of the next injection [17]. She initiated pumping on the unaffected right breast to stimulate increased production and continued to provide the infant with additional formula supplementation as needed. At the advice of her breast surgeon, she started galactagogues with brewer’s yeast and moringa three times daily. She used ibuprofen and acetaminophen as needed for pain, as well as warm compresses and ice. Her milk production on the left breast was low, and she did not require hand expression to relieve engorgement.

On follow up 3 weeks later, the patient had developed right nipple pain. This pain was considered related to increased engorgement in the right breast, as well as, the infant clamping in adjustment to increased flow of unilateral milk. The patient was instructed in lymphatic massage and utilized a laid-back breastfeeding position to reduce velocity of flow in the right breast [18, 19].

Her left breast pain had improved, and the mass had decreased in size to 6.0 × 6.0 × 5.0 cm from 11:00–3:00. She had developed a new draining fistula in the 1:00 position. An additional 40 mg/1 mL triamcinolone mixed with 3 mL 2% lidocaine was injected into the affected areas of her left breast.

**Fig. 1:**
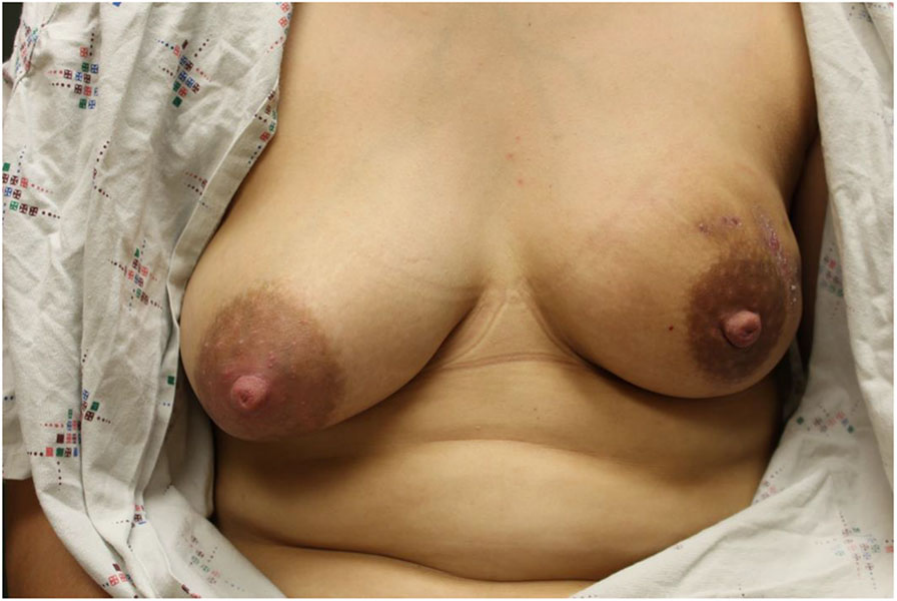
At 10 weeks postpartum, the left breast affected by IGM is no longer lactating; right breast is lactating

**Fig. 2:**
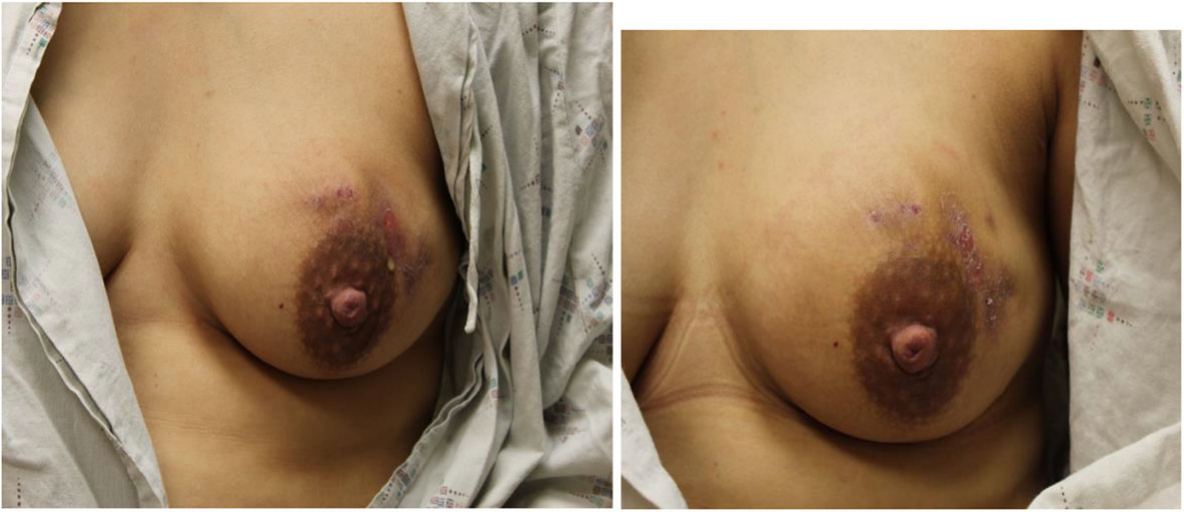
**a** Left breast affected by IGM at week 3 after presentation (7 weeks postpartum) and (**a**) Left breast affected by IGM at week 6 after presentation (10 weeks postpartum)

At 10 weeks postpartum (6 weeks after presentation to the breast surgeon), the patient indicated that she would like to attempt to resume breastfeeding from the affected left breast. On examination, the fistula was closed and there was a decreased mass-like area in the left breast to approximately 4.0 × 3.0 × 2.0 cm size. Therefore, no steroid injection was performed. The images showing the difference in volume between her right and left breasts at this time is demonstrated in Fig. 1. Comparison images demonstrating improvement in her left breast are illustrated in Fig. 2.

Three weeks later, the patient presented with increased redness and pain in the previously affected left breast. The patient had attempted breastfeeding from the left breast, but had stopped due to pain and low milk production. A new fistula in the 1:00 position and developed with new erythema and fluctuance in the 7:00 position with an associated new 2.0 × 2.0 × 1.0 cm mass-like area. An injection of 40 mg/1 mL triamcinolone mixed with 3 mL 2% lidocaine was performed into the 1:00 and 7:00 positions of the left breast and she was advised to stop breastfeeding from the left breast after the injection.

At three-week follow-up, the patient had developed a new 9:00 position fistula, but there was decreased mass-like effect and erythema in the remainder of the breast. She elected to resume breastfeeding from the left breast and start and oral prednisone taper over 6 days, *30 mg of prednisone per day, tapering to 5 mg per day*. She noticed gradual decrease in erythema and fluctuance and no new fistulae. She continued to breastfeed from both breasts until 7 months postpartum. At this point, she stopped breastfeeding due to gradual decrease in milk production over time.

## Discussion and conclusions

### Presentation

In all patients, whether lactating or not, IGM is a challenging clinical scenario to manage, particularly since many women experience a protracted waxing and waning course of symptoms. Some patients may present with a mass alone that spontaneously regresses, whereas others demonstrate significant erythema, fluid collection formation, and recurrent fistulization from the onset of the disease [20].

Most symptomatology is localized to the periareolar region extending outward [21]. As one area of inflammation becomes more quiescent, new disease may present in a new quadrant of the breast [20]. Without any intervention, the IGM generally will resolve within a few years of presentation [22]. With systemic immunosuppression with methotrexate and/or azathioprine, the disease tends to subside earlier. However, patients may experience a flare months after apparent resolution [10, 23]. Importantly, azathioprine has a good safety profile in pregnancy and lactation [24].

### Evaluation and diagnosis

All patients with suspected IGM require breast imaging and core needle biopsy to confirm diagnosis; this is safe in breastfeeding [2]. Tuberculosis should be ruled out [25] and patients should be referred to a rheumatologist to rule out underlying autoimmune disorder. The relationship with erythema nodosum and IGM is unclear, but it has been hypothesized that it can represent an extra-mammary form of IGM [26].

### General principles of treatment

In the non-lactating population, traditional treatment has included systemic steroids, methotrexate and antibiotics, local care involves procedures ranging from incision and drainage, wide local excision with wound vacuum placement, or mastectomy [2, 27]. A systematic review of 3060 patients found that more industrialized countries use surgery more frequently, and more rural countries have a tendency to rely more heavily on antibiotics [28]. Regardless of location, steroids are often used, and it is common for IGM to require a combination of several therapies [28]. A large study of IGM that investigated 206 individuals in Iran recommends corticosteroids as first line treatment for this disease [3]. There are reports of surgical management with improved cosmetic outcomes and minimal complications, including mastectomy with immediate reconstruction, but there is also report of surgery causing increased inflammatory reaction and fistulazation [7, 10, 29]. While anti-tuberculosis agents such as rifampin have been utilized, they also have been associated with rebound inflammation [30, 31].

Cultures of fluid collections often demonstrate *Corynebacterium, Streptococci*, *Staphylococci* or *Bacteroides*; however, the traditionally utilized antibiotics of doxycycline and trimethoprim-sulfamethoxazole have not shown clinically significant results in terms of disease severity or duration [32, 33]. This is likely related to the thought that IGM is an inflammatory rather than infectious disease, and culture results reflect the presence of common breast flora [34]. More recently, IGM has been thought to have a significant autoimmune component, which supports the notion that localized steroids could be an effective treatment strategy [31].

Surgical intervention also remains controversial. While one study demonstrates a shortened duration to resolution compared with steroid injections and observation alone, not all cases require such aggressive treatment [9]. More recently, it has become clear that repeated surgical intervention can potentiate inflammation and can increase disease severity [35]. One group compared recurrence for individuals who were treated with antibiotics and wide local excision, or incision and drainage plus steroids. The less invasive approach had significantly less recurrence [35]. A retrospective study that demonstrated excision had a lower recurrence rate than the comparison group. However, this study was limited in that the comparison underwent warm compress, herb application, and drainage alone; this conservative approach without steroids would not be expected to treat the disease effectively [36].

Instead of traditional invasive approaches that have not demonstrated clear success, intralesional triamcinolone injections every 1–2 weeks represent a promising minimally invasive approach with limited systemic side effects and good outcomes [37]. After the first steroid injection, patients experience a marked decrease in disease volume, symptoms, and frequency of recurrence [37]. Additionally, patients who receive steroid injections were likely to achieve a complete recovery without the adverse effects of oral steroids [37]. The frequency of injection can be adapted based on clinical circumstances, such as severity of presentation.

While triamcinolone injection directly into breast lesions can relieve symptoms, due to the potential for infant exposure to the steroid via breast milk, this treatment modality is not compatible with continued breastfeeding on the treated breast(s). This is related to high oral dose to infant and the potential infant side effects of growth suppression, and interference with corticosteroid production [17]. The procedure for intralesional injection is outlined below, and can be performed with or without ultrasound guidance with an 18-gauge needle.

- Clean skin
- Aspirate any existing fluid
- Mix 40 mg/ 1 mL triamcinolone with 3 mL 2% lidocaine for a total volume of 4 mL
- Inject deeply into areas of active fistula, mass, erythema or induration.

Wound care should consist of managing drainage from fistulae with gauze and other non-adherent dressings. Tape should be avoided due to further abrasion and irritation of skin. Patients often experience significant distress related to the symptoms of the disease and psychosocial support should be provided.

### Treatment during lactation

There is no data to suggest that breastfeeding prolongs the duration of symptoms of IGM, and the diagnosis of IGM does not preclude lactation. Interestingly, a recent study demonstrated a small minority of IGM patients, not in the lactational period, who have demonstrated elevated prolactin from either dopamine antagonist medications or a prolactinoma [38]. Like much of other proposed pathophysiologic mechanisms in IGM, the exact relationship to prolactin remains unclear.

Special considerations for treatment for breastfeeding women include the fact that oral steroids decrease milk production [39]. Maternal insomnia, agitation, and risk for depression also are of concern in the postpartum population [39, 40]. Triamcinolone injections in the breast are contraindicated with breastfeeding from that breast for approximately 2 weeks [17]. In high doses such as those used with anti-neoplastic therapy, methotrexate is not compatible with breastfeeding. Limited information suggests that single or weekly doses may demonstrate low levels of methotrexate in breastmilk, but some expert opinions also warn against this use during lactation [41]. Azathioprine as studied primarily in inflammatory bowel disease populations, is safe during lactation, may provide more ideal systemic therapy in the breastfeeding population [42].

We recommend individualized care with considerations given to the following options:

- Continuing oral steroids with knowledge oral steroids may not be particularly effective. Systemic steroids cause known side effects as outlined above, including reduction in milk production, insomnia, agitation, and depression, however low-dose or even higher doses for a short duration are likely compatible with lactation [39].
- Avoiding use of methotrexate, which is generally incompatible with lactation. Consideration of azathioprine, which is compatible with lactation.
- Stopping breastfeeding and pumping from the affected breast, augmenting production on the unaffected breast, and utilizing intralesional triamcinolone injections on the affected breast.
- Not utilizing steroids and managing patients with supportive care only, such as gauze for draining fistulae to keep skin dry and minimize maceration.
- Pain management including non-steroidal anti-inflammatory medications such as ibuprofen and acetaminophen, as well as conservative management with ice and heat per patient comfort.
- Repeated antibiotic treatments are not effective and can result in unintended and avoidable side effects, such as *Clostridium difficile* infections, gastrointestinal distress and future resistance.
- Psychosocial support for patient distress and discomfort
- Lactation consultant (IBCLC) involvement or other breastfeeding specialist to assist in unilateral weaning, to assure adequate milk supply and to offer breastfeeding support.

In conclusion, IGM is a challenging chronic inflammatory breast disease that affects women primarily in the reproductive years, with a higher incidence in patients of Hispanic, Native American, Middle Eastern, and African descent. Traditional treatment has included systemic immunosuppression and surgery, but newer literature demonstrates that intralesional injection of steroid can provide significant symptomatic relief to patients. A diagnosis of IGM does not preclude breastfeeding, though patients may experience challenges with milk production and latch on the affected breast. Individualized care should be provided, with considerations given to the side effects of systemic steroids; the need to wean a breast being treated with intralesional steroids; and, augmentation of milk production on the unaffected breast to promote continued breastfeeding.

## Data Availability

Not applicable.

## Abbreviations

IGM: Idiopathic Granulomatous Mastitis
NAC: Nipple Areolar Complex
